# Effect of portal vein ligation on tumor growth and liver regeneration in rat cirrhotic liver lobes

**DOI:** 10.3892/etm.2014.1607

**Published:** 2014-03-06

**Authors:** RUI XU, YU-FENG YUAN, AHMET AYAV, CHONG-QING JIANG, LAURENT BRESLER, ZHI-SU LIU, NGUYEN TRAN

**Affiliations:** 1 General Surgery Department, Zhongnan Hospital of Wuhan University, Wuhan, Hubei 430071, P.R. China; 2 School of Surgery, Faculty of Medicine-UHP, University of Nancy, Nancy 54501, France

**Keywords:** portal vein ligation, tumor growth, liver regeneration, cirrhosis

## Abstract

The aim of the present study was to investigate the effect of portal vein ligation (PVL) on the tumor growth rate and liver regeneration in rat cirrhotic liver lobes. A total of 45 male Wistar rats were randomly divided into PVL, hepatic tumor (HT) and HT + PVL groups (n=15 per group). Liver regeneration and tumor growth in ligated and non-ligated lobes were evaluated prior to and following PVL. In addition, serum alanine transaminase, total bilirubin levels and liver tissue samples were evaluated. The results indicated that PVL induced apparent hypertrophy in normal and HT rats. However, the ratio of non-ligated lobes to total liver weight or body weight in the HT + PVL group was significantly lower when compared with the PVL group (P<0.05). Compared with the HT group, the tumor growth rate in the ligated lobes of the HT + PVL group significantly increased (P<0.05). However, tumor growth in the non-ligated lobes exhibited no statistically significant difference between the HT and HT + PVL groups. In addition, Knodell scores indicated that fibrosis was more apparent in the non-ligated lobes of the HT + PVL group when compared with the HT group (P<0.05). Therefore, tumor growth was accelerated in ligated lobes following PVL, but not in non-ligated lobes. PVL also induced liver regeneration in cirrhotic liver lobes with lower efficiency than that in the non-cirrhotic lobes. However, hypertrophy in the contralateral cirrhotic lobes appeared to be non-functional.

## Introduction

Liver resection is the only curative treatment for a number of patients with primary or secondary liver tumors (1). Portal vein occlusion is widely used to induce liver hypertrophy in the future remnant liver (FRL) prior to major liver resection (2). Two strategies are available to induce hypertrophy of the liver: Portal vein ligation (PVL) and portal vein embolization (PVE). Although a number of clinicians consider PVE to be superior to PVL, previous studies have shown that PVL is as effective as PVE in inducing hypertrophy to the volume of the remnant liver (3,4). Preoperative portal vein occlusion by PVE or PVL is an effective method to increase the volume of the FRL. While the use of PVE and PVL is increasing, there is growing evidence that PVE and PVL stimulate not only the growth of the FRL, but also affect tumor size in occluded and non-occluded liver segments (5,6).

The prevalence of cirrhosis in patients with hepatocellular carcinoma (HT) is between 80 and 90%, while 10–20% of HT cases develop in patients without cirrhosis (7). At present, it is commonly accepted that PVL and PVE are generally safe procedures that have few side effects in non-cirrhotic patients (8). Sakai *et al* (6) demonstrated that PVL accelerates tumor growth in ligated lobes, but not contralateral lobes. However, to date, there have been no studies specifically demonstrating the effect of PVE or PVL on liver tumor growth and regeneration in cirrhotic liver lobes. Thus, in the present study, a rat model of PVL was used to determine the effects of ligation on HT growth and liver regeneration. In addition, the association between cirrhosis severity and tumor growth was evaluated in ligated and non-ligated lobes.

## Materials and methods

### Animal preparation

Male Wistar rats weighing 350–400 g were purchased from the Center for Animal Experiment/Animal Bio-safety Level III Laboratory of Wuhan University (Wuhan, China) and the Animal Facility of Nancy University (Nancy, France). The Research Committees of the two universities approved the animal experimental procedures. All the rats received standardized care in accordance with the National Institutes of Health Guidelines for Ethical Animal Research. Animals were maintained in an animal experimental room at a temperature of 25±5°C under a 12-h light/dark cycle.

### Study protocol

A total of 45 rats were randomly divided into three groups. The PVL group consisted of normal rats that received PVL (n=15). The HT group consisted of rats with tumors that did not receive PVL (n=15) and the HT + PVL group comprised rats with tumors that received PVL (n=15). Rats in the HT and HT + PVL groups were fed a diet containing diethylnitrosamine (DEN; Sigma Aldrich, St. Louis, MO, USA; 95 mg/kg body weight/week) for 12 weeks to induce hepatocellular carcinomas. Rats that survived with evident HTs were selected for further study. Surgery was performed when the administration of DEN was completed. PVL was performed in the PVL and HT + PVL groups, while the rats in the HT group received sham-operation. Blood (at days 0, 1, 2 and 7) and liver (at day 14) samples were collected for analysis following surgery. Tumor size was measured using positron emission tomography (PET) scans 7 days prior to PVL, as well as at day 7 and 14 following PVL. Ligated and non-ligated liver lobes were also measured at day 14 following PVL. The tumor growth rate and ratios of non-ligated lobe weight to total body weight and non-ligated lobe weight to ligated lobe weight were calculated.

### PVL

Rats were anesthetized via inhalation of isoflurane/O_2_ (Baxter International, Inc., Munich, Germany). Following a midline laparotomy, the liver was removed from the ligaments. A double running suture was performed on sham-operated rats. The selective PVL was performed on the middle and left lobes (lobes 1–3) under a microscope. Then, the middle and left lobes (lobes 1–3) were defined as ligated lobes, while the right lobes were defined as non-ligated lobes (lobes 4–7). The corresponding portal veins of the liver were ligated with a 7-0 Fumalen following careful dissection of the hepatic artery. Portography was performed prior to and following the selective PVL to visualize the liver anatomy and to demonstrate portal occlusion of the appropriate liver segments.

### Blood analysis

Blood samples were obtained from the femoral vein and centrifuged at 3,000 × g for 10 min at 4°C. Blood serum was stored at −20°C prior to analysis. Alanine transaminase (ALT) and total bilirubin (TBI) levels were measured using a TBA-2000FR System (Toshiba Corporation, Tokyo, Japan).

### Evaluation of tumor size and liver weight

Tumor size was measured using PET 7 days prior to PVL and at day 7 and 14 following PVL. Rats were sacrificed 2 weeks following PVL and the weights of the ligated and non-ligated lobes were measured using a laboratory microscale (Sartorius AG, Goettingen, Germany). The ratios of non-ligated lobe weight to total body weight and non-ligated lobe weight to ligated lobe weight were calculated. The tumor growth rate was calculated using the following formula: Growth rate = (TV2 − TV1)/TV1, where TV1 was the tumor volume prior to PVL and TV2 was the tumor volume following PVL.

### Histological examination

Morphological examination was performed before and 14 days after PVL. Liver biopsy specimens were obtained from the rats prior to PVL for histological evaluation and all the rats were sacrificed at day 14 following PVL for histological evaluation. Under a light microscope, three observational fields were randomly selected in each specimen and were blindly evaluated by two pathologists following randomization. Liver tissue was assessed in each case using a modified Knodell scoring system via four main aspects on hematoxylin and eosin (H&E) stained sections: Periportal and bridging necrosis, intralobular degeneration, focal necrosis, portal inflammation and fibrosis.

### Statistical analysis

Data are expressed as the mean ± SEM. Comparisons among mean values were performed by one- or two-way analysis of variance. Statistical analysis was conducted using SPSS software 8.0 (SPSS, Inc., Chicago, IL, USA) and a two-tailed value of P<0.05 was considered to indicate a statistically significant difference.

## Results

### Changes in ALT and TBI serum levels

**Figure 1:**
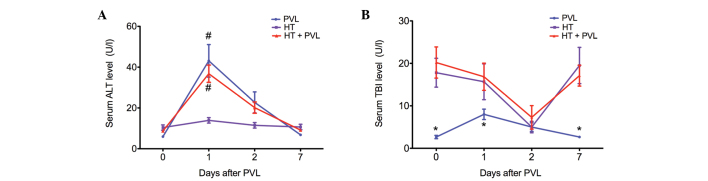
Serum levels of (A) ALT and (B) TBI (mean ± SEM). Day 0 indicates the time point prior to PVL. ^#^P<0.05, vs. other time points; ^*^P<0.05, vs. other groups. ALT, alanine transaminase; TBI, total bilirubin levels; PVL, portal vein ligation.

Serum levels of ALT were assessed as a measure of hepatocyte necrosis, while TBI levels were analyzed to determine whether the biliary tract was injured during the PVL surgery (Fig. 1). In the PVL and HT + PVL groups, serum ALT levels were significantly increased at day 1 and 2 following PVL (P<0.05). However, TBI levels in the PVL and HT + PVL groups did not increase until 2 days after PVL surgery. In addition, TBI levels in the HT group were significantly higher as compared with those in the PVL group at day 0, 1 and 7 (P<0.05). However, there was no statistically significant difference in TBI levels between the HT and HT + PVL groups (P>0.05).

### Liver growth and regeneration following PVL

**Figure 2:**
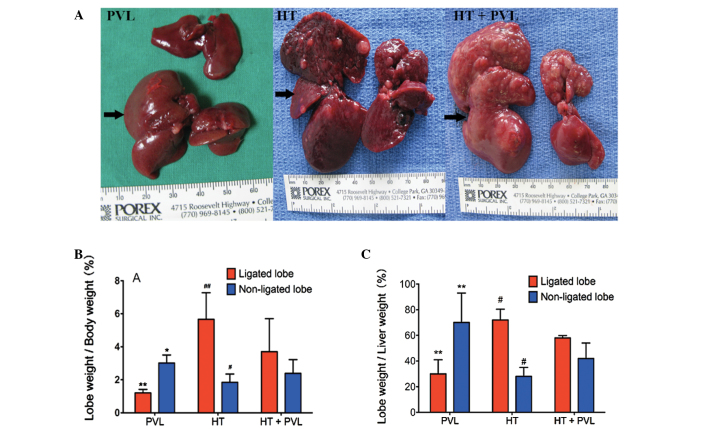
Ligated and non-ligated lobes in different groups (A) and changes in the ratios of non-ligated and ligated lobe weights to (B) body weight and (C) total liver weight following PVL. Ligated lobes in the HT group were referred to as lobes 1–3, while non-ligated lobes in the HT group were referred to as lobes 4–7. Black arrows indicate the non-ligated lobes in the PVL and HT + PVL groups or lobes 4–7 in the HT group. ^*^P<0.05 and ^**^P<0.01, vs. other groups; ^#^P<0.05 and ^##^P<0.01, vs. HT + PVL group. Data are expressed as the mean ± SEM (n=15 per group). HT, hepatic tumor; PVL, portal vein ligation.

To evaluate liver growth and regeneration following PVL, ratios between non-ligated or ligated lobe weights to total body weight and non-ligated lobe weight to total liver weight were measured two weeks following PVL surgery. As shown in Fig. 2, the ratio of ligated lobe weight to total body weight in the HT + PVL group was significantly higher as compared with the PVL group (P<0.01), while the ratio of non-ligated lobe weight to total body weight in the HT + PVL group was significantly lower as compared with the PVL group (P<0.05). In addition, the ratio of ligated lobe weight to total liver weight in the HT + PVL group was significantly lower as compared with the PVL group (P<0.01). Compared with the non-ligated lobes in the HT + PVL group, the ratios of lobes 4–7 to total body weight and to total liver weight were significantly lower in the HT group (P<0.05).

### Tumor growth following PVL

**Figure 3:**
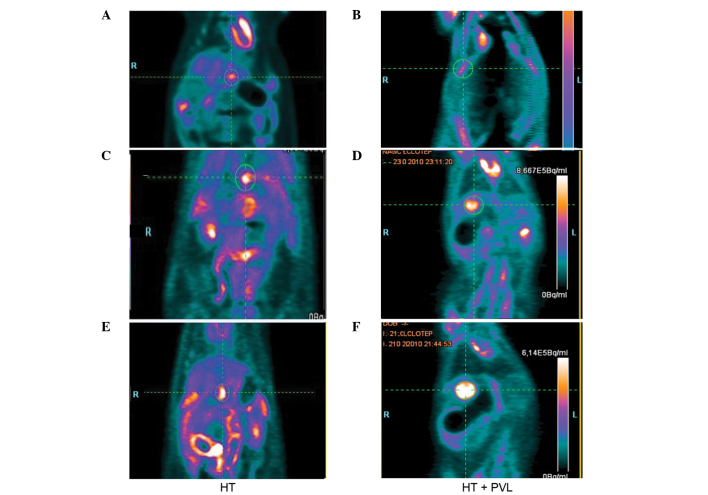
Tumor size evaluation with PET scans in the ligated lobes at (A and B) prior to PVL, (C and D) day 7 following PVL and (E and F) day 14 following PVL. Ligated lobes in the HT group were referred to as lobes 1–3. PET, positron emission tomography; PVL, portal vein ligation; HT, hepatic tumor.

**Figure 4:**
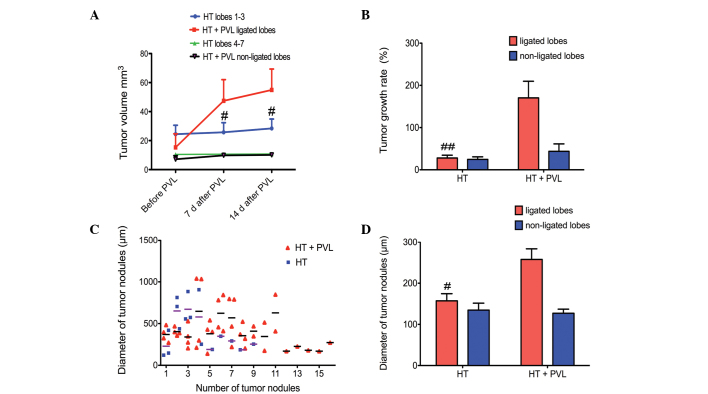
Changes in tumor growth in the ligated and non-ligated lobes. (A) Changes in tumor size prior to and following PVL. (B) Tumor growth rate in the ligated and non-ligated lobes. (C) Number of tumor nodules and their various diameters. (D) Average diameter of the tumor nodules in the ligated and non-ligated lobes. ^#^P<0.05 and ^##^P<0.01, vs. HT + PVL group. HT, hepatic tumor; PVL, portal vein ligation.

Tumor size was evaluated by PET scans prior to PVL and at day 7 and 14 following PVL surgery (Fig. 3). With regard to the non-ligated lobes, tumor size in the HT + PVL group was similar to that of lobes 4–7 in the HT group (P>0.05; Fig. 4). However, tumor size in the ligated lobes of the HT + PVL group significantly increased following PVL surgery and was significantly higher when compared with the lobes 1–3 of the HT group (P<0.05; Fig. 4A). The tumor growth rate in the ligated lobes of the HT + PVL group was significantly higher than that of lobes 1–3 in the HT group (P<0.01; Fig. 4B). There was no significant difference in the tumor growth rate between the non-ligated lobes of the HT + PVL group and lobes 4–7 of the HT group (P>0.05). The number of tumor nodules was counted in the HT and HT + PVL groups. In addition, the diameters of the tumor nodules were measured under a light microscope. The average diameter of the tumor nodules in the ligated lobes of the HT + PVL group was significantly higher when compared with lobes 1–3 of the HT group (P<0.05; Fig. 4C and D). However, there was no significant difference between lobes 4–7 of the HT and HT + PVL groups.

### Histological evaluation in ligated and non-ligated lobes

**Figure 5:**
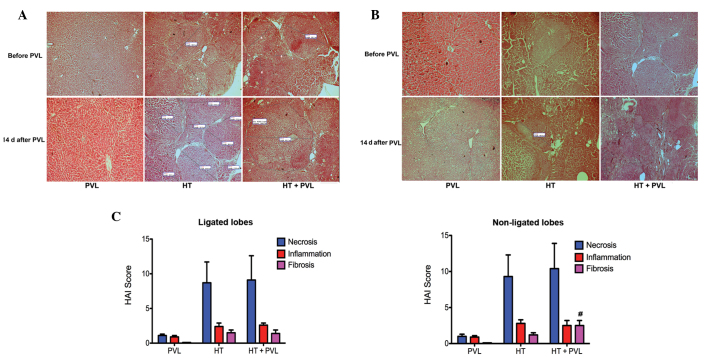
Histological evaluations of H&E stained sections of (A) ligated and (B) non-ligated lobes (magnification, ×200). (C) Knodell Histology Activity Index scores for the ligated and non-ligated lobes, based on the combined scores for necrosis, inflammation and fibrosis. ^#^P<0.05, vs. other groups. H&E, hematoxylin and eosin.

Following administration of DEN for 12 weeks, the cirrhosis severity varied among the rats in the HT + PVL and HT groups. Liver tissue was assessed in each case with H&E stained sections. Fig. 5 shows the liver histology of the rats prior to and two weeks following PVL. In the HT and HT + PVL groups, numerous tumor nodules were observed in the ligated and non-ligated lobes under a light microscope (Fig. 5A and B). Varying degrees of necrosis, intralobular degeneration, portal inflammation and fibrosis were observed in the samples from the HT and HT + PVL groups prior to PVL surgery. Histological evaluation prior to PVL revealed no injuries in the liver of the rats in the PVL group. Hypertrophy was induced by PVL in the non-ligated lobes of the PVL and HT + PVL groups. Following PVL surgery, connective tissues accumulated and reticulin fibers spread radially throughout the liver in the ligated lobes of the HT + PVL group. Compared with the HT group, the number of infiltrating inflammatory cells in the liver insignificantly increased in the HT + PVL group and the deposition of fibrous components around the portal area also increased. According to the Knodell index, hepatic fibrosis in the non-ligated lobes of the HT + PVL group was more apparent than that in the lobes 4–7 of the HT group (P<0.05; Fig. 5C). These results indicate that PVL promotes the onset of hepatic fibrosis during hypertrophy formation in non-ligated cirrhotic lobes.

## Discussion

In the current study, the effects of PVL on tumor growth and liver regeneration were evaluated in ligated and non-ligated cirrhotic liver lobes. The changes in serum ALT and TBI levels indicated that PVL was successfully conducted. The results demonstrated that hypertrophy in non-ligated lobes was apparent in normal and HT rats. In addition, the tumor growth rate in the ligated lobes increased following PVL surgery, however, in the non-ligated lobes, there were no marked changes following surgery. The liver regeneration rate in non-ligated lobes and degeneration rate in ligated lobes was much higher in the normal rats (PVL group) than in the HT rats (HT + PVL group). Furthermore, PVL promoted the onset of hepatic fibrosis during hypertrophy formation in the non-ligated cirrhotic lobes.

At the beginning of the 20^th^ century, non-ligated lobe regeneration was recognized following portal branch ligation. As previously reported, PVL can be achieved safely without causing mortality and is an effective method to induce hypertrophy (3,5). In the present study, PVL surgery successfully induced hypertrophy in normal and HT rats. However, liver regeneration in the normal rats was much more apparent when compared with the HT rats. Significantly, histological evaluation revealed that the increased contralateral lobes primarily consisted of fibrous tissue and tumor nodules in the cirrhotic livers following PVL. Therefore, hypertrophy in cirrhotic liver lobes may be considered as non-functional.

Compensatory hyperplasia is possibly stimulated by hepatotrophic substances that are contained in portal blood flow or by increased blood flow in the non-occluded portal vein branch (9,10). In addition, it is commonly accepted that liver regeneration depends predominantly on the proliferation of hepatocytes (11–13). As previously reported, the incidence of cirrhosis or fibrosis is high in primary liver cancer (14). Liver cirrhosis is characterized by diffuse disorganization of the normal hepatic structure of regenerative nodules and fibrotic tissue. Consequently, decreased numbers of hepatocytes may result in a lower regenerative ability. In addition, cirrhosis leads to portal hypertension and hyperdynamic circulation that can have widespread effects in the body (15). Endothelial dysfunction is generally observed among cirrhotic patients with portal hypertension (16). However, a previous study demonstrated that inductive angiocrine signals from the sinusoidal endothelium are required for liver regeneration (17). Other studies have also reported that vascular endothelial growth factor promotes liver regeneration by increasing the proliferation of hepatocytes (18,19).

There are a number of indications from clinical and experimental studies that, despite liver atrophy, tumors in ligated lobes do not shrink in size, but rather show acceleration of growth (2,6,8,20). The observations of the present study are consistent with these studies that have demonstrated increased tumor growth in ligated lobes following PVL. A previous study demonstrated that accelerated tumor growth appeared to be a result of increased growth factor expression (6). Following PVL surgery, the expression levels of tumor necrosis factor (TNF)-α and interleukin (IL)-6 were significantly higher in the ligated lobes compared with the non-ligated lobes. TNF-α and IL-6 have been implicated as important contributors to liver growth and regeneration (11,21). Increased hepatocyte growth factor (HGF) and epidermal growth factor (EGF) levels may be an additional explanation for the accelerated tumor growth due to the stimulatory effects that HGF and EGF exhibit on tumor cells (6,22).

The present study has several limitations. Firstly, a rat model was used to investigate the effects of PVL on tumor growth and liver regeneration. Compared with humans, rats differ with regard to anatomy and physiology. Secondly, the present study did not offer any insight into the potential mechanisms that contribute to the histological changes in the non-ligated cirrhotic liver lobes. Thus, further studies are required to investigate the underlying mechanisms.

In conclusion, the results of the present study support the hypothesis that PVL accelerates tumor growth in ligated lobes, but not in contralateral lobes. In addition, the results indicate that PVL induces liver regeneration in cirrhotic liver lobes with lower efficiency than in non-cirrhotic lobes. Hypertrophy in the contralateral cirrhotic lobes is predominantly a consequence of hepatic fibrosis. Thus, PVL for cirrhotic liver lobes should be considered carefully in the future work.
